# A fluorescence-based protocol to monitor bacterial antagonism

**DOI:** 10.1128/spectrum.03183-24

**Published:** 2025-06-12

**Authors:** Justin M. Luu, Cristian V. Crisan, Morgan L. Pettis, Anayancy Ramos Facio, Timothy D. Read, Joanna B. Goldberg

**Affiliations:** 1 Microbiology and Molecular Genetics Program, Graduate Division of Biological and Biomedical Sciences, Laney Graduate School, Emory University 310203 , Atlanta, Georgia, USA; 2 Division of Pulmonary, Asthma, Cystic Fibrosis, and Sleep, Department of Pediatrics, Emory University School of Medicine 209740, Atlanta, Georgia, USA; 3 Emory+Children’s Center for Cystic Fibrosis and Airway Disease Research, Emory University School of Medicine 12239, Atlanta, Georgia, USA; 4 Department of Biology, Emory University 123382, Atlanta, Georgia, USA; 5 Division of Infectious Diseases, Department of Medicine, Emory University School of Medicine 234195, Atlanta, Georgia, USA; The Ohio State University College of Dentistry, Columbus, Ohio, USA

**Keywords:** bacterial competition, interspecies interactions, bacterial killing protocol

## Abstract

Bacteria are often found in polymicrobial communities where competition for limited space and resources drives antagonistic interactions. Therefore, bacteria have evolved various antibacterial weapons to outcompete their neighbors. These antagonistic interactions can have profound effects on the structure, dynamics, and composition of bacterial communities. To study interactions between two bacterial species, co-culture assays are often employed. The most common approach utilizes selection plates for each bacterial species to quantify recovery after co-incubation. Although this method is relatively accurate and inexpensive, there are some limitations. These assays can be time-consuming, low-throughput, and may present difficulties if the bacteria of interest show similar resistance patterns to one another or if the tolerance to selective agents is unknown. Here, we have developed and validated a method that uses fluorescence as a proxy to screen antibacterial interactions between two species. We utilized two fluorescently tagged bacteria, *Staphylococcus aureus* JE2 and *Escherichia coli* DH5⍺, both expressing a red fluorescent protein (RFP), and competed them against non-fluorescent bacteria: two strains of *Pseudomonas aeruginosa* or one strain of *Stenotrophomonas maltophilia*. We observed that RFP production correlates with growth in the reporter strains and that a reduction in relative fluorescent units from the reporter strains corresponds with a reduction in colony-forming units. This method is fast, semi-quantitative, semi-high-throughput, and can be used to rapidly screen for antagonistic activity during bacterial co-cultures. We propose that our protocol can be a useful tool to detect antibacterial activity using fluorescently labeled target bacteria.

**IMPORTANCE:**

In nature, bacteria often reside in communities where limited space and resources drive competition. Bacterial antagonistic interactions can profoundly affect microbial communities. A common approach to study these interactions is to measure the recovery of each bacterium after competition by using selective media. While relatively accurate and inexpensive, this approach has a few limitations: the assay can be labor-intensive and time-consuming, is low throughput, and can present issues when the bacterial strains of interest have similar antimicrobial resistance or if their resistance profile is unknown. We developed and validated a fast and semi-high-throughput protocol that gauges antagonistic bacterial interactions using fluorescence as a proxy. As proof of principle, this screening protocol was tested with known antagonistic bacteria, using a fluorescently labeled target bacterium.

## INTRODUCTION

Bacteria rarely live in isolation and are often found in polymicrobial communities (1, 2). In humans, polymicrobial communities exist throughout the body and are collectively known as the microbiome (3). These polymicrobial communities can be associated with infections found in various sites of the human body, such as the skin, teeth, wounds, and lungs (4). Infections caused by these bacterial communities can lead to increased complications, such as altered tolerance to antibiotic treatments or increased virulence compared to infections with single species (5, 6).

In polymicrobial communities, bacteria must compete for space and resources. Therefore, they often engage in antagonistic interactions, which can be mediated by diffusible products (like antimicrobials, toxins, or bacteriocins) or contact-based weapons (like protein secretion systems) (7–11). These antagonistic interactions can have profound effects on the structure, dynamics, and composition of bacterial communities (8, 12, 13).

Multiple methods have been used to assess interactions between two bacterial species. The most common approach involves co-incubation of bacteria, followed by plating onto media that selects for the bacterial species of interest, and counting surviving colony-forming units (CFUs). The extent of competition can be expressed as a ratio of CFUs of the bacteria when mixed in co-culture compared to the individual bacteria grown alone. This method has the advantage of being relatively accurate and inexpensive. Our group used this colony-counting framework to survey the interactions of clinical isolates of *Staphylococcus aureus*, *Pseudomonas aeruginosa,* and *Stenotrophomonas maltophilia* (14–17). For studies with *S. aureus* and *P. aeruginosa*, we previously co-cultured 64 *S*. *aureus* isolates with *P. aeruginosa* as well as 28 co-isolated pairs of *S. aureus* and *P. aeruginosa* after they were grown together on agar plates (14, 15). These studies highlighted some limitations related to this method as it is generally labor-intensive, difficult to scale, and time-consuming (a co-culture experiment can take about 5–7 days from start to finish). Using selection plates is a less practical solution when attempting to determine the potential of tens or hundreds of bacterial isolates to engage in antagonistic interactions. Another limitation is that, while selection medium for *S. aureus* and *P. aeruginosa* co-cultures has been well defined (14, 15), it may be problematic if the competing bacteria are resistant to multiple antibiotics (and if this resistance is also found in the target bacteria), or if the antibiotic resistance is not known. Other media, such as differential media, can also be used to separate species but present an issue if these media cannot distinguish between the bacteria of interest. Therefore, a reliable method to quickly screen the antagonistic potential of multiple bacterial isolates is required.

**Fig 1:**
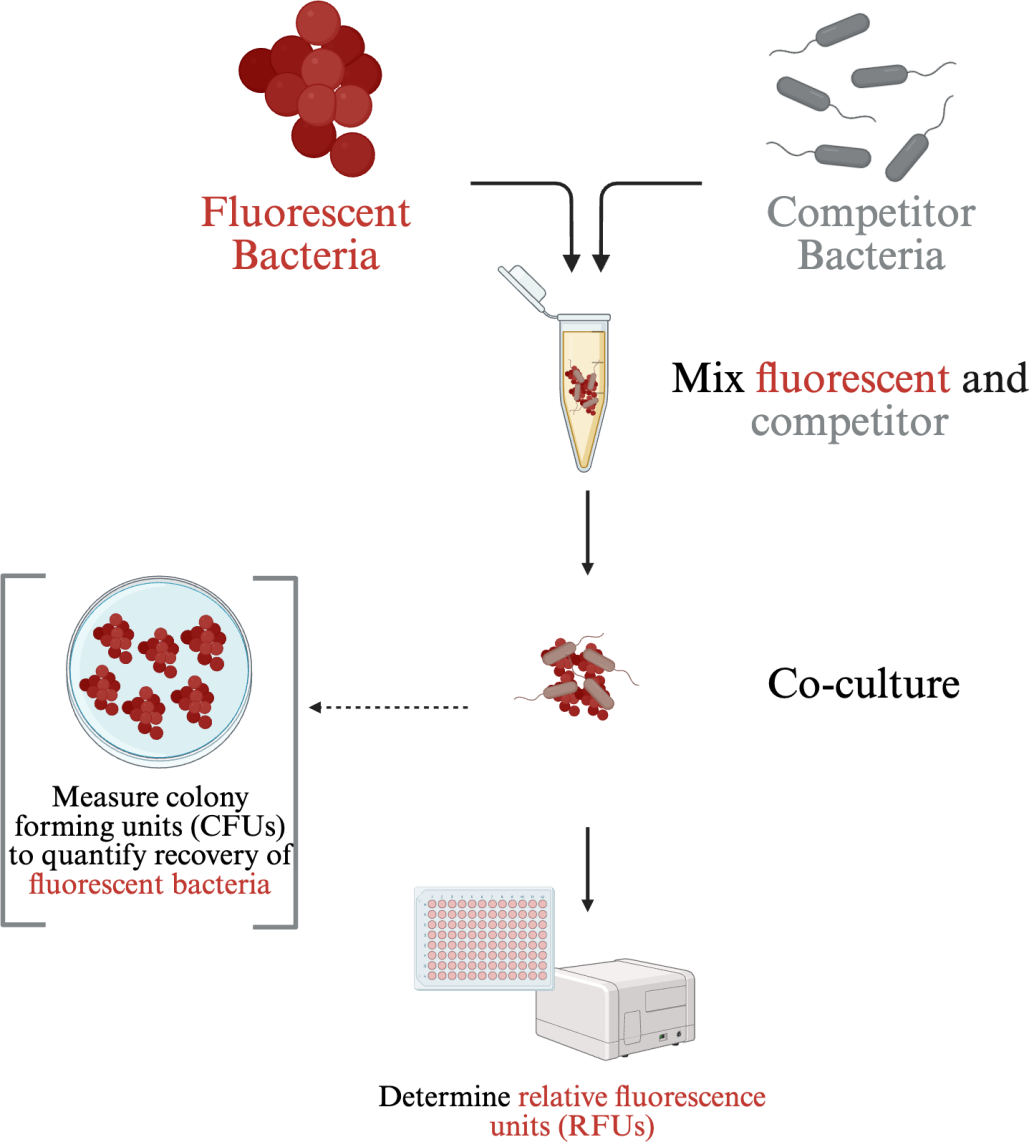
Schematic representation of the fluorescence-based protocol. To test antagonistic activity against the target bacteria, the potential competitor is mixed with the fluorescently labeled target bacteria. The mixture is then plated on an agar plate or inoculated in liquid media. After co-culture, relative fluorescence units (RFUs) are determined in a plate reader. Colony-forming units (CFUs) were also determined on selection agar for each species to validate the assay. Figure was generated in BioRender.

Here, we developed and validated a simple method to assess antibacterial potential using fluorescence as a proxy (Fig. 1). To test this approach, we used two fluorescently tagged target bacteria: *S. aureus* JE2 expressing DsRed red fluorescent protein (RFP) and *Escherichia coli* DH5⍺ strain expressing a mCherry RFP as target bacteria (18, 19). We then competed these fluorescently labeled reporter strains with two bacterial species often isolated from polymicrobial infections: *P. aeruginosa* and *S. maltophilia* (15, 17, 20). These four bacterial species were chosen as they have been known to be isolated from respiratory infections in patients with cystic fibrosis, can be isolated together, and their interactions have been studied. Antagonistic behaviors between *P. aeruginosa* and *S. aureus* have been well described, and *P. aeruginosa* has been shown to antagonize *E. coli* as well (21–25). Furthermore, our group has shown that *S. maltophilia* has multiple antibacterial weapons and can antagonize both *S. aureus* and *E. coli* (10, 16, 17). We demonstrate that our method can be utilized to screen for antibacterial interactions and can be adapted for a larger number of bacterial isolates, as it does not require a selective medium.

## RESULTS

### Reporter strains show significantly higher fluorescence than competitor bacteria

**Fig 2:**
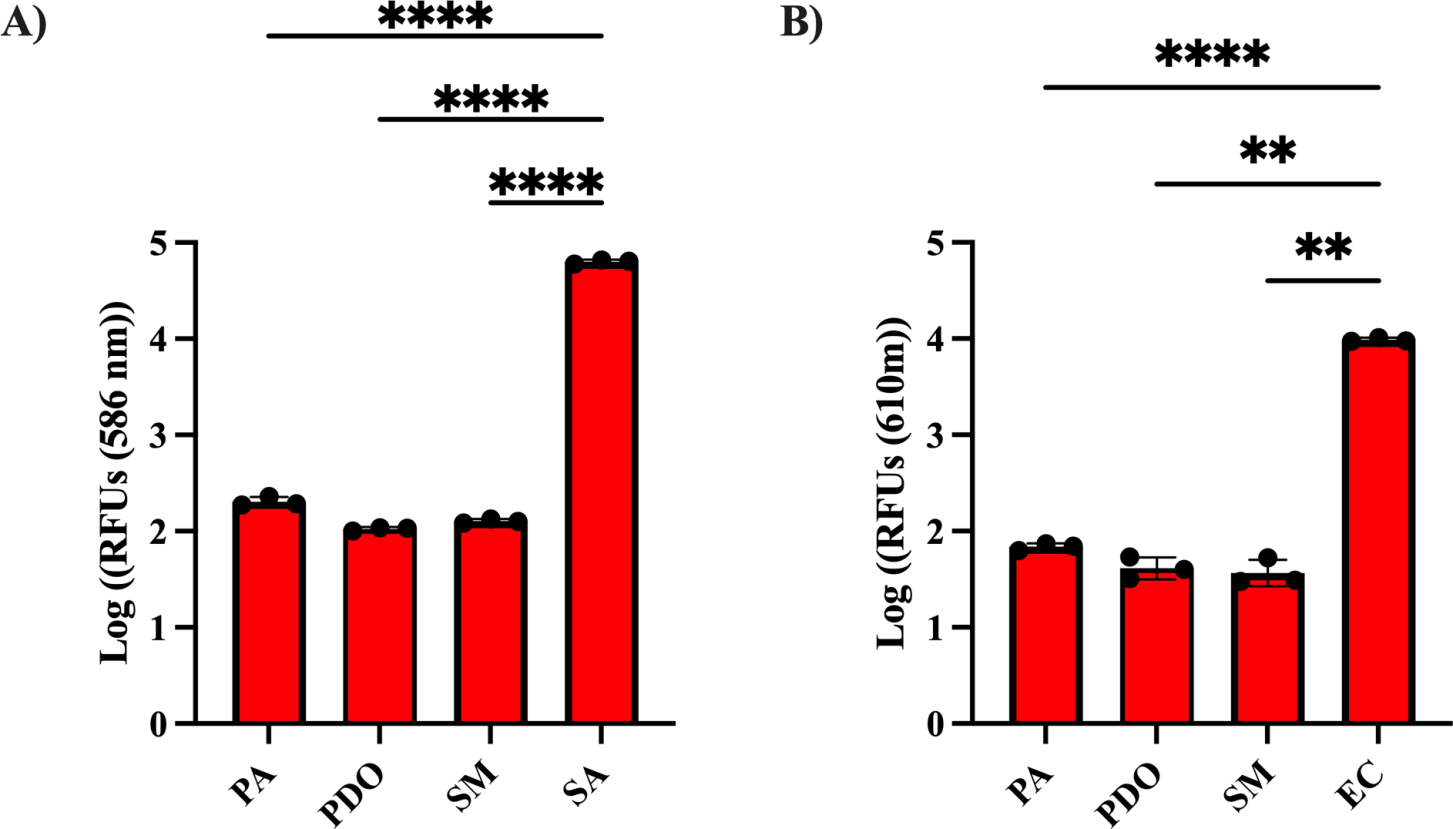
Reporter strains show significantly higher fluorescence than competitor bacteria. To determine the autofluorescence of each strain relative to the reporters, each strain was plated on an LA plate at a starting OD_600_ of 0.05. Relative fluorescence units (RFUs) of each competitor strain were measured and compared to SA (**A**) and EC (**B**). Fluorescence for SA was measured by DsRed production (554 nm excitation and 586 nm emission wavelength), and EC was measured by mCherry (587 nm excitation and 610 nm emission wavelength). The means of three biological replicates and standard deviations are shown. Significance was determined using a one-way ANOVA with Dunnett’s multiple comparisons test. ****P < 0.0001 and **P < 0.01.

Since our protocol only utilizes fluorescence to probe antagonistic potential, one consideration is that bacterial species may have intrinsic levels of fluorescence (referred to as autofluorescence). To determine whether the amount of autofluorescence emitted from competitor strains would affect our protocol, we measured relative fluorescence unit (RFU) levels for monocultures of competitor and reporter strains (described in Table 1). We observed that *S. aureus* JE2 expressing DsRed red fluorescent protein (referred to as SA) and *E. coli* DH5⍺ strain expressing a mCherry RFP (referred to as EC) displayed significantly greater fluorescence compared to each of the competitor bacteria (Fig. 2A and B). Overall, these results suggest that fluorescence can be used to distinguish between reporter and competitor strains.

**TABLE 1:** Strains used in this study^*a*^

Abbreviation	Strain	Species	Description	Plasmid	Reference
SA	JE2-DsRed	*Staphylococcus aureus*	Gram-positive bacteria fluorescent reporter	pHC48	(18)
EC	DH5⍺-mCherry	*Escherichia coli*	Gram-negative bacteria fluorescent reporter	pUC20T	(19)
PA	PAO1	*Pseudomonas aeruginosa*	Competitor bacteria	n/a	(15)
PDO	PDO300	*Pseudomonas aeruginosa*	Competitor bacteria	n/a	(15)
SM	CCV131	*Stenotrophomonas maltophilia*	Competitor bacteria	n/a	(17)

^*a*^ n/a, not applicable.

### RFP production in the reporter strains correlates with growth in liquid media

**Fig 3:**
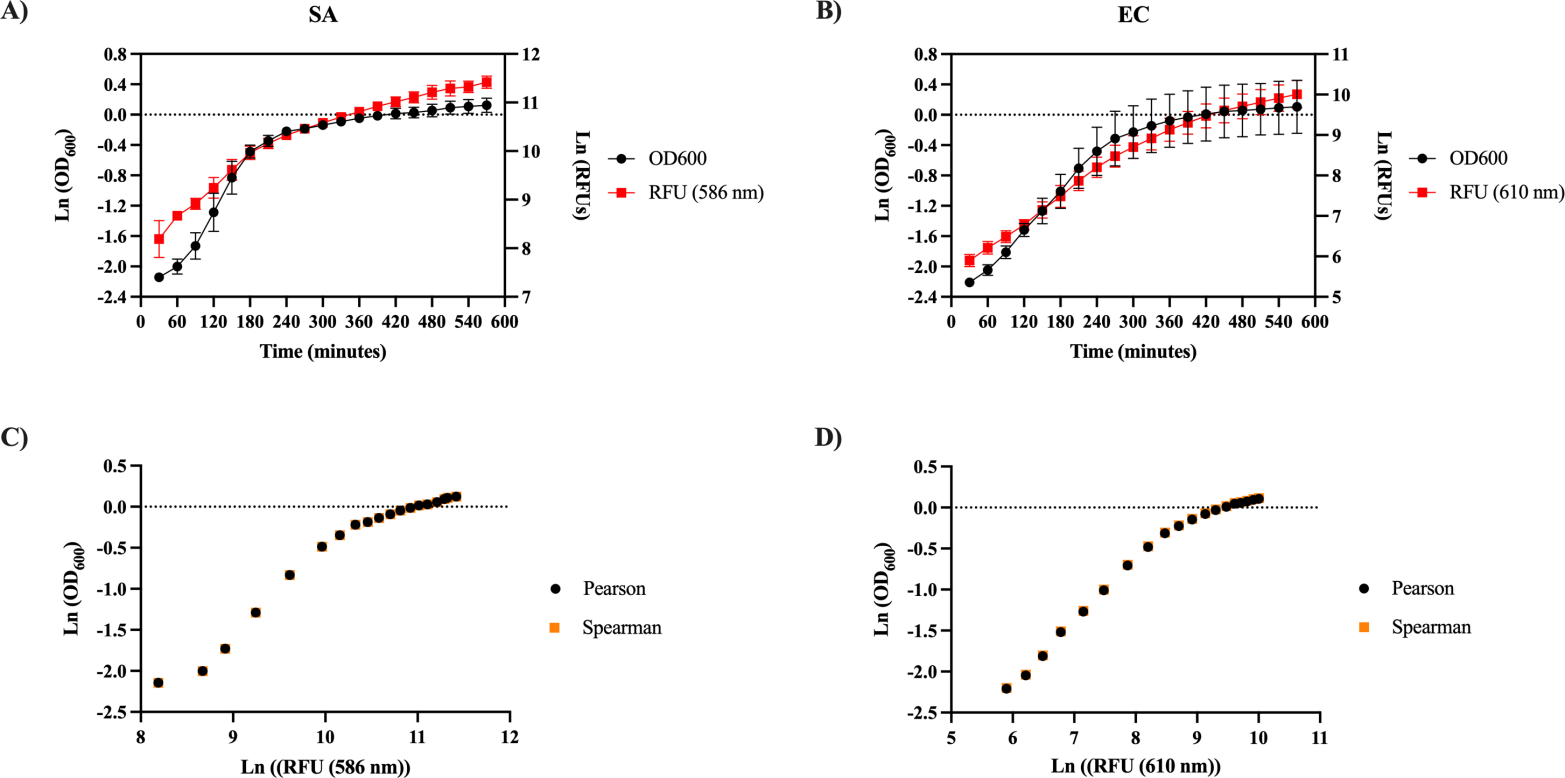
Reporter strain growth corresponds to red fluorescent protein production. SA (**A**) and EC (**B**) monocultures were grown in a 96-well plate in lysogeny broth at a starting OD_600_ of 0.05. OD_600_ and relative fluorescence units (RFUs) were measured every 30 minutes for 570 minutes in a plate reader. Changes in OD_600_ and RFUs are represented in black and red, respectively. OD_600_ and RFUs were converted to natural logarithmic values, and the mean of six biological replicates and the standard deviation are shown. The correlation between OD_600_ and RFU was calculated with both the Pearson (black) and Spearman (orange) correlation coefficients for SA (**C**) and EC (**D**).

Previous studies have explored how different fluorophores can be used to gauge bacterial interactions (26–28). We first sought to determine how RFP production correlates with bacterial growth. Each reporter strain was cultured in a 96-well microtiter plate, and both OD_600_ and RFP values were measured every 30 minutes (Fig. 3). As expected, bacterial growth followed a sigmoid curve, while RFP production gradually increased over time, but with a delayed response. Despite the slightly different dynamics of the OD_600_ and RFP production, we observed a strong correlation between bacterial growth and the natural logarithm of RFP for both SA (Pearson coefficient *r*^2^ = 0.95 and *P* < 0.0001, Spearman coefficient *r* = 1 and *P* < 0.0001) (Fig. 3C) and EC (Pearson coefficient *r*^2^ = 0.96 and *P* < 0.0001, Spearman coefficient *r* = 1 and *P* < 0.0001) (Fig. 3D).

### A reduction in RFUs in the reporter strains corresponds with a reduction in CFUs when co-cultured with competitor strains

**Fig 4:**
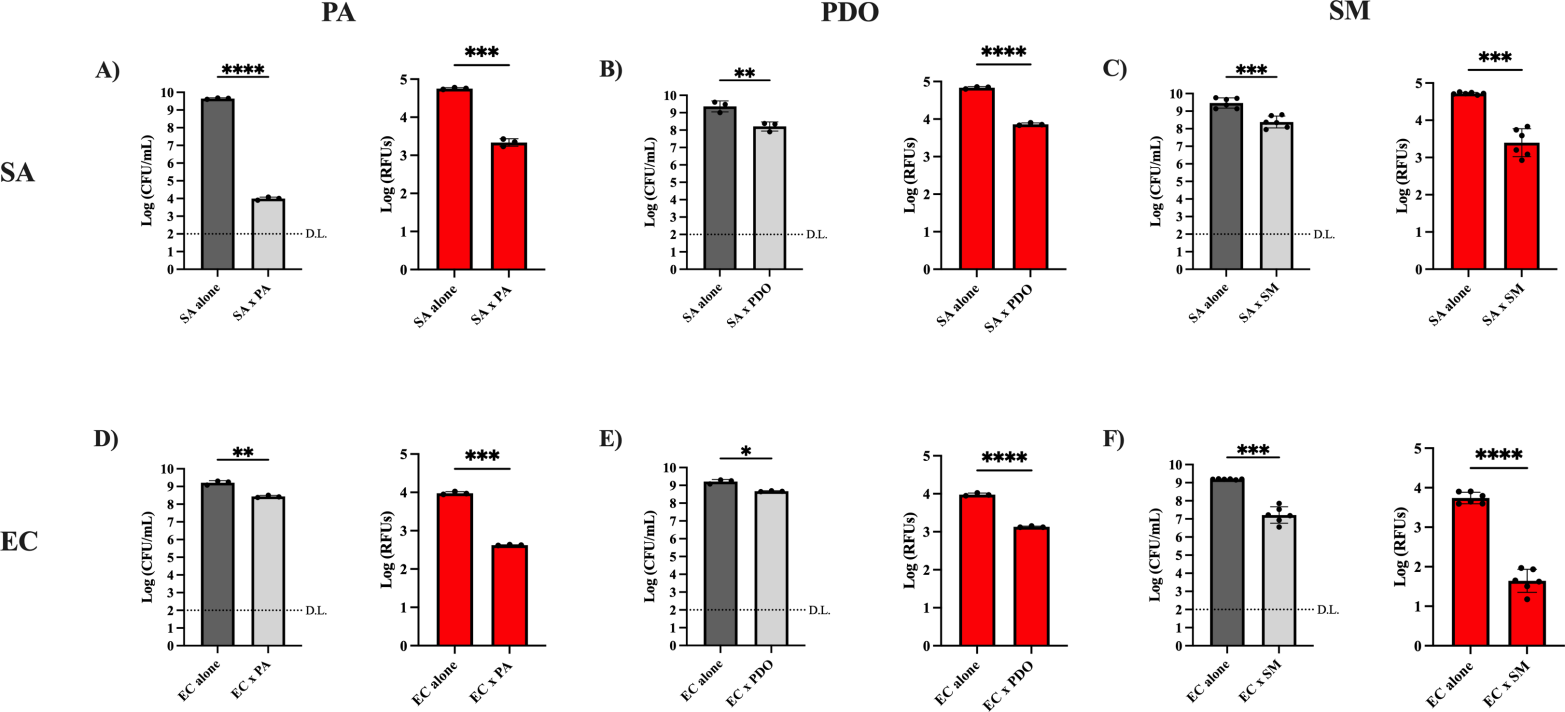
Co-culturing with the competitor strains result in a reduction in both CFUs and RFUs. SA was co-cultured at a 1:1 ratio with PA (**A**), PDO (**B**), and SM (**C**) at a starting OD_600_ of 0.05 on LA plates for 24 hours. EC was also co-cultured with PA (**D**), PDO (**E**), and SM (**F**) following the same conditions. RFUs and CFUs of the co-cultures were determined and compared to monoculture controls. The means of at least three biological replicates and standard deviations are displayed. D.L., limit of detection. Statistical significance was determined by an unpaired *t*-test with Welch’s correction. ****P < 0.0001, ***P < 0.001, **P < 0.01, and *P < 0.05.

We next sought to demonstrate that fluorescence can be used to measure the survival of target cells. We co-cultured each reporter strain with the competitor strains *P. aeruginosa* PAO1, *P. aeruginosa* PDO300, or *S. maltophilia* CCV131 (referred to as PA, PDO, or SM, respectively) (Table 1). For these initial studies, reporter and competitor strains were competed at a 1:1 ratio at a starting OD_600_ of 0.05 on agar plates for 24 hours. We observed a decrease in RFUs for both SA and EC (Fig. 4) when co-cultured with the competitor bacteria, which corresponded with a decrease in CFUs. These results indicate that RFUs can be used as a proxy to gauge antibacterial interactions with both reporter strains under the conditions tested.

Next, we performed co-cultures of each reporter strain with competitor strains under different ratios (reporter:competitor) and different starting optical densities. First, we modified the ratio of reporter to competitor bacteria to either 1:10 or 10:1 (Fig. S1A through C and S2A through C). Next, we tested the original 1:1 ratio of reporter to competitor but at a higher starting OD_600_ of 1 (Fig. S1D through F and S2D through F). Finally, we tested modifying the ratio of reporter to competitor bacteria to either 1:10 or 10:1 at a starting OD_600_ of 1 (Fig. S1G through I and S2G through I). With SA, a decrease in CFUs recovered corresponded with a decrease in RFUs under each of these conditions tested. For EC, we observed that the trend was consistent across different ratios when competed at a starting OD_600_ of 0.05 and at a 1:1 ratio at a starting OD_600_ of 1. When EC was competed with PA and PDO at a 10:1 ratio and at a starting OD_600_ of 1, the decrease in EC CFUs was not statistically significant compared to monocultures; however, the recovered RFUs were lower. In all cases, we observed that a decrease in CFUs corresponds with a decrease in RFUs. Overall, these results validate that RFUs from the reporter strains can be used as a tool to gauge outcomes of antibacterial interactions.

**Fig 5:**
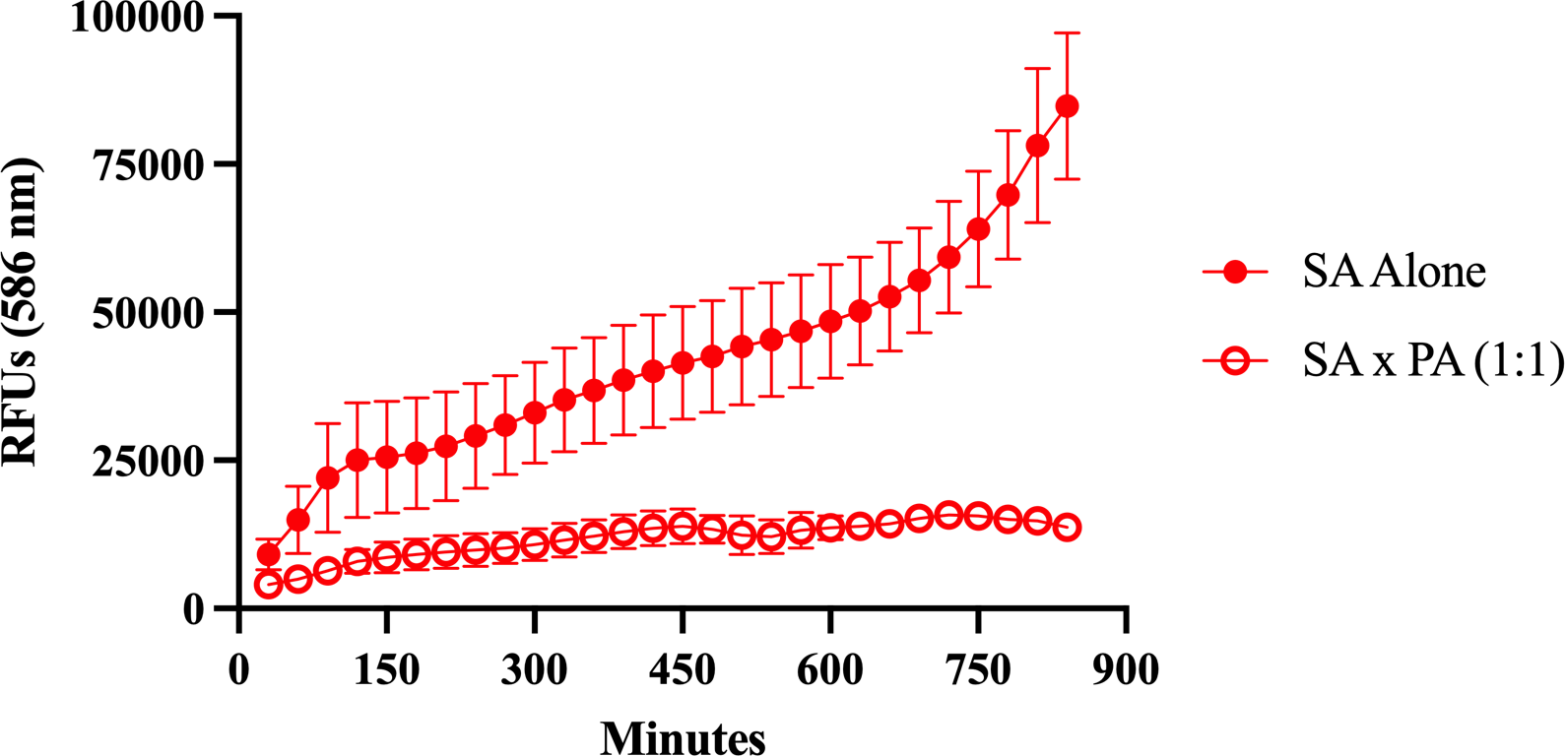
SA RFP signal is reduced when co-cultured with PA in liquid. SA and PA were competed at a 1:1 ratio with a starting OD_600_ of 0.01 in TSB. RFUs were measured every 30 minutes for 14 hours in 96-well plates. The mean of three biological replicates, with three technical replicates each, and the standard deviation are shown.

To further validate the application of this protocol in other commonly used methods to study antagonistic interactions, we chose one co-culture condition to test in liquid media. We tested SA and PA at a 1:1 ratio, grown in tryptic soy broth (TSB) at a starting OD_600_ of 0.01. We observed that the presence of PA led to a reduction in RFP signal during co-cultures compared to SA grown alone (Fig. 5). These results are consistent with our observations on solid media and indicate that our protocol can be applied to studying bacteria on both solid and liquid media.

## DISCUSSION

Here, we developed and verified a fluorescence-based method to screen for antibacterial interactions between multiple species of interest against a reporter strain. Since fluorescence is read immediately after co-culture, it removes the need to serially dilute, plate out each condition, incubate plates and wait for colonies to form, and then count colonies. It is easier to scale up the number of co-cultures performed as fluorescence can be measured in 96-well plates, increasing the number of conditions that can be tested per experiment.

We validated the protocol using both gram-positive and gram-negative reporter strains with different fluorophores to highlight the versatility of this method. By validating our protocol using different bacterial reporters, we have shown that this method can be applied to a wide variety of bacteria. The use of different fluorophores also indicates that this protocol is not limited to one specific fluorophore.

While there are other published methods to monitor bacterial competition, our protocol provides specific advantages depending on the application. Lin and Lai (27) developed a protocol that measures bacterial competition on a high-throughput scale in a 96-well plate. This method provides a more quantitative measurement compared to our protocol; however, its efficiency is dependent on the use of a pipetting robot, and competition is measured by CFUs on antibiotic selection plates. Smith and Septer (26) developed a protocol that quantifies bacterial competition at a single-cell level using fluorescence. This protocol can directly measure the number of bacteria recovered in competition; however, it is limited in its ability to scale up to the number of bacteria tested. Taillefer et al. (28) developed a protocol that measures bacterial competition through the release of β-galactosidase. While this protocol and ours both measure bacterial competition through fluorescence, their choice of reporter strains is limited to bacterial species that can produce β-galactosidase. Our protocol utilizes plasmids that can be easily transformed into different species. Alseth et al. (29) studied polymicrobial communities and determined the abundance of each microbe through qPCR using species-specific primers. Our protocol provides the advantage of being able to measure the effects of a polymicrobial community on a reporter strain without the need to design primers and perform expensive techniques like qPCR. Overall, the strength of our protocol lies in its simplicity and its ability to quickly and easily screen a large collection of bacteria, such as clinical and environmental strains, against reporter strains. Finally, it is also sensitive to both bacteriostatic and bactericidal antagonism.

There are a few limitations with our protocol that should be noted. First, we observed a delay in the production of fluorescence when compared to the growth of each reporter strain. One explanation for this increased delay could be that the rate of RFP production may not directly match the doubling rate of each unlabeled reporter. Another explanation could be that it takes time to accumulate enough RFP to be detected via fluorescence. We also noticed that RFU values produced by SA monocultures were approximately six times the RFU values produced by EC monocultures. This could be due to the differences in plasmid copy numbers or promoter strength. Regardless of these factors, the RFUs produced by the reporters were orders of magnitude greater than each competitor strain. For each new pair of strains to be competed, we recommend checking the baseline level of fluorescence of the reporter strains and competitors before proceeding with experiments, as some subtle differences may be missed. Another limitation is that while a decrease in CFUs does correspond with a decrease in RFUs, it does not measure the exact number of surviving cells. In its current form, this protocol only measures fluorescence at an endpoint. However, it can easily be adapted to take multiple measurements, especially if bacteria are co-cultured together in liquid in a microtiter plate.

We propose that our RFP protocol can be best adapted as a tool to screen for pairs of competing bacteria to further study. While this protocol was verified with set parameters, it can be modified based on the intended application. We have validated this approach in liquid media and saw the expected decrease in RFP signal when SA was co-cultured with PA compared to SA monocultures. While not directly tested here, this protocol could be useful for monitoring the survival of the reporter strains in a polymicrobial environment with more than one competitor. Similarly, it could be adapted to test for antibacterial activity, as has recently been described using luciferase-expressing reporters (30). Fluorophore production could also be used to track the viability of the competitor strain in tandem with the reporter strain. By inserting different fluorophore-expressing plasmids in both the competitor and reporter, one could study how interactions impact either strain. In conclusion, we have developed an efficient protocol that utilizes fluorescent reporters to gauge antibacterial interactions between species.

## MATERIALS AND METHODS

### Bacterial strains and culture conditions

Five bacterial strains (two reporter strains and three competitor strains) were utilized in this study (Table 1). A *S. aureus* JE2 strain expressing DsRed from the pHC48 plasmid (SA) and an *E. coli* DH5⍺ strain expressing mCherry from the pUC20T plasmid (EC) were used as gram-positive and gram-negative reporter strains, respectively. pHC48 constitutively expresses DsRed under the *S. aureus sarA* P1 promoter, is maintained by chloramphenicol, and has a pBR322 origin of replication (18). mCherry is constitutively expressed in pUC20T under a *lacZ* promoter, maintained by carbenicillin, and has a pBR322 origin of replication (19). The competitor strains were *P. aeruginosa* PAO1 (referred to as PA), *P. aeruginosa* PDO300 (referred to as PDO), and *S. maltophilia* CCV131 (referred to as SM); we have previously reported how PA and PDO antagonize *S. aureus* JE2 (15) and how SM competes with *E. coli* DH5⍺ (17). Prior to the co-culture experiments on solid media, strains were grown on lysogeny broth agar plates (LA) at 37°C overnight. Liquid lysogeny broth (LB) cultures, supplemented with antibiotics when appropriate, were inoculated with single colonies and grown overnight at 37°C. For liquid co-culture experiments, PA was grown on LA plates and SA was grown on tryptic soy agar plates overnight at 37°C. Single colonies of these strains were inoculated into TSB supplemented with antibiotics, when appropriate, before being grown overnight at 37°C with shaking.

### Autofluorescence measurements

Overnight cultures of all strains were diluted 1:50 in fresh LB and grown for 3 hours at 37°C. Antibiotics were supplemented to the growth media for strains carrying plasmids. Strains were washed with LB and adjusted to an optical density (OD_600_) of 0.05. A volume of 5 µL of each strain was spotted onto LA plates, plates were allowed to dry, and then incubated at 37°C for 22 hours. Cultures were aseptically excised and vortexed for at least 30 seconds in 1 mL of phosphate-buffered saline (PBS). A volume of 200 µL of each culture was plated on a Costar black 96-well plate, and RFUs for each reporter were read.

### Growth curves

Overnight cultures of the indicated strains were diluted to an OD_600_ of 0.05 in fresh LB. A volume of 200 µL of each culture was added to a Costar black 96-well plate. The plate was then incubated in a Biotek Synergy H1 Hybrid Reader and grown at 37°C with double orbital shaking. OD_600_ and RFUs for each strain were measured every 30 minutes for 570 minutes.

### Plate co-culture experiments

Overnight cultures of all strains were diluted 1:50 in fresh LB and grown for 3 hours at 37°C. Antibiotics were supplemented to the growth media for strains carrying plasmids. Strains were washed with LB and adjusted to an OD_600_ of either 0.05 or 1. For co-cultures with PA or PDO, a 1× volume of competitor bacteria was mixed with either a 1× or 10× volume of each reporter strain. For co-cultures with SM, a 1× or 10× volume of SM was mixed with a 1× volume of each reporter strain. Each reporter strain was mixed with either 1× or 10× volume of LB as a mono-culture control. A volume of 5 µL of each mixture was spotted onto a LA plate, allowed to dry, and incubated for 22 hours in a 37°C incubator. The mixtures were then aseptically excised, transferred to a 15 mL culture tube with 1 mL of PBS, and vortexed for at least 30 seconds. A volume of 200 µL of each culture was added into the well of a Costar black 96-well plate, and RFUs were read. They were then serially diluted and plated on selection agar plates for the reporter species. SA was selected on *Staphylococcus* isolation agar (LB + 7.5% NaCl). EC was selected on M63 minimal medium to select against SM or LA plates supplemented with carbenicillin (100 µg/mL) to select against PA and PDO. The CFUs were determined the following day.

### Liquid co-culture experiments

Overnight cultures of SA were washed and resuspended in fresh TSB. Resuspended SA and overnight PA cultures were then diluted to an OD_600_ of 0.01 in fresh TSB. Liquid co-culture experiments were then prepared by mixing SA and PA to a 1:1 ratio, and 200 µL of this mixture was plated in a Costar Black 96-well plate. Both PA and SA were also grown alone as mono-culture controls. Cultures were then grown for 14 hours at 37°C with double orbital shaking, with fluorescence and OD_600_ readings captured every 30 minutes.

### Fluorescent measurement parameters

For all fluorescent measurements, samples were loaded into a Costar black 96-well plate with a clear bottom (Corning) and read in a Biotek Synergy H1 Hybrid Reader (Agilent). Fluorescence was measured from the bottom of the plate at a set gain of 100. Strains with DsRed were measured at an excitation wavelength of 554 or 560 nm and an emission wavelength of 586 nm. Strains with mCherry were measured at an excitation wavelength of 587 nm and an emission wavelength of 610 nm.

### Statistical analyses

All statistical analyses were performed using GraphPad Prism 9. Correlations between growth and fluorophore production were calculated using both the Pearson and Spearman correlation coefficients. The statistical significance between each group was calculated with either a one-way ANOVA with Dunnett’s multiple comparison test or an unpaired Student’s *t*-test with Welch’s correction when appropriate.

## SUPPLEMENTAL MATERIAL

Supplemental figures spectrum.03183-24-s0001.docx Fig. S1 and S2.

ASM does not own the copyrights to Supplemental Material that may be linked to, or accessed through, an article. The authors have granted ASM a non-exclusive, world-wide license to publish the Supplemental Material files. Please contact the corresponding author directly for reuse.
