# Supplementary material for: A fluorescence-based protocol to monitor bacterial antagonism
Source: Microbiol Spectr. 2025 Jun 12;13(8):e03183-24. doi: 10.1128/spectrum.03183-24 (PMC12323326; doi:10.1128/spectrum.03183-24)
Supplement: Supplemental figures — Fig. S1 and S2. [file spectrum.03183-24-s0001.docx]

**SUPPLEMENTAL MATERIAL**

**Spectrum03183-24R1**

**A Fluorescence-Based Protocol to Monitor Bacterial Antagonism**

Justin M. Luu, Cristian V. Crisan, Morgan L. Pettis, Anayancy Ramos Facio, Timothy D. Read, and Joanna B. Goldberg

**SA x Competitor Strains Under Various Conditions**

**
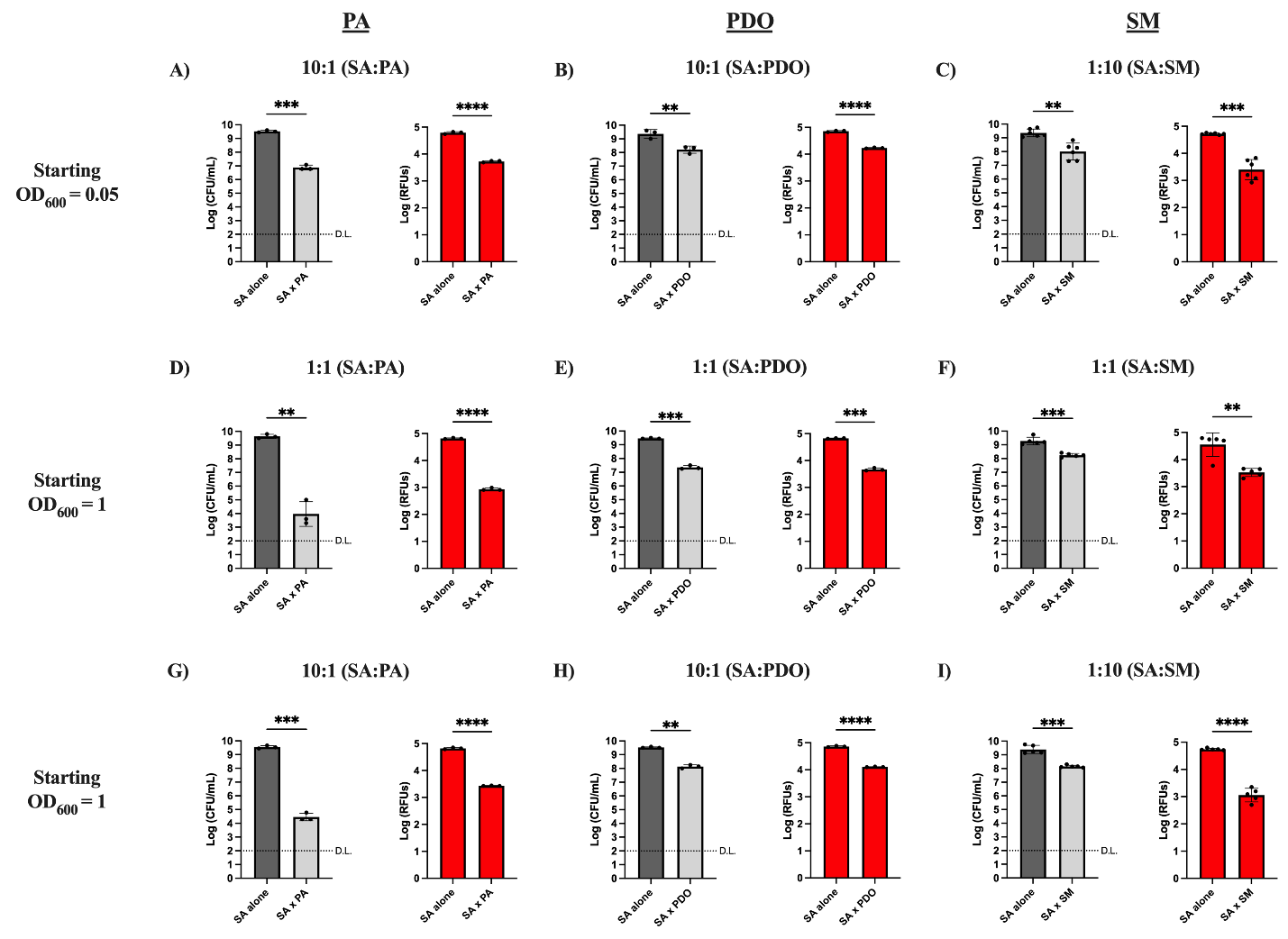
**

**Supplemental figure 1. A reduction in RFUs in SA corresponds to a reduction in CFUs when co-cultured the competitor strains under various conditions.** SA was co-cultured with PA (left column), PDO (middle column), and SM (right column) at different starting OD_600_ and ratios. A starting OD_600_ of 0.05 was tested at different ratios of reporter to competitor: 10:1 (reporter: competitor) ratio with PA (A), PDO (B) and 1:10 with SM (C). The reporters and competitors were then tested at a higher starting OD_600_ of 1 at a 1:1 ratio (D, E, F). At the higher starting OD_600_ of 1, different ratios of reporter to competitor were also tested: 10:1 (reporter: competitor) ratio with PA (G), PDO (H) and 1:10 with SM (I). RFUs and CFUs of the co-cultures were determined and compared to monoculture controls. The mean of three to six biological replicates and standard deviations are displayed. D.L. - limit of detection. Statistical significance was determined by an unpaired t-test with Welch’s correction. **** p < 0.0001, *** p < 0.001, ** p < 0.01.

**EC x Competitor Strains Under Various Conditions**


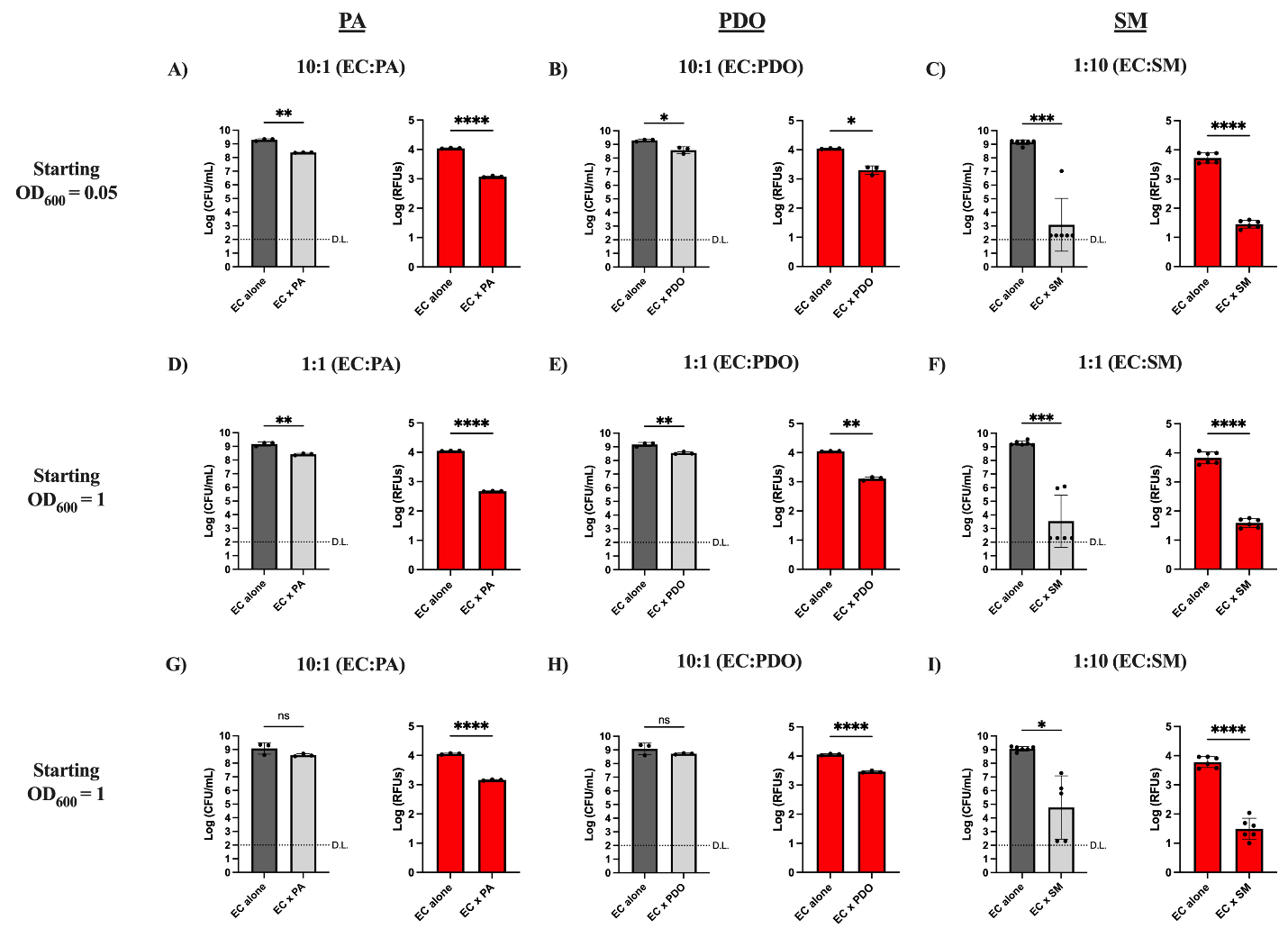


**Supplemental figure 2. A reduction in RFUs in EC corresponds to a reduction in CFUs when co-cultured with competitor strains under various conditions.** EC was co-cultured with PA (left column), PDO (middle column), and SM (right column) at different starting OD_600_ and ratios. A starting OD_600_ of 0.05 was tested at different ratios of reporter to competitor: 10:1 (reporter: competitor) ratio with PA (A), PDO (B) and 1:10 with SM (C). The reporters and competitors were then tested at a higher starting OD_600_ of 1 at a 1:1 ratio (D, E, F). At the higher starting OD_600_ of 1, different ratios of reporter to competitor were also tested: 10:1 (reporter: competitor) ratio with PA (G), PDO (H) and 1:10 with SM (I). RFUs and CFUs of the co-cultures were determined and compared to monoculture controls. The mean of three to six biological replicates and standard deviations are displayed. D.L. - limit of detection. Statistical significance was determined by an unpaired t-test with Welch’s correction. **** p < 0.0001, *** p < 0.001, ** p < 0.01, * p < 0.05.
